# Parasitism of *Arapaima gigas* (Schinz, 1822) in fish farms of the state of Amazonas, Brazil

**DOI:** 10.1590/S1984-29612022064

**Published:** 2022-12-12

**Authors:** Marilson Farias Gama, Daniel Brito Porto, Hélio Daniel Beltrão dos Anjos, Franmir Rodrigues Brandão, Gabriela Tomas Jerônimo, Patricia Oliveira Maciel-Honda, Edsandra Campos Chagas

**Affiliations:** 1 Programa de Pós-graduação em Ciência Animal e Recursos Pesqueiros – PPGCARP, Universidade Federal do Amazonas – UFAM, Manaus, AM, Brasil; 2 Instituto Federal de Educação, Ciência e Tecnologia do Amazonas – IFAM, Lábrea, AM, Brasil; 3 Instituto Nacional de Pesquisas da Amazônia – INPA, Manaus, AM, Brasil; 4 Secretaria Municipal de Educação, Manaus, AM, Brasil; 5 Embrapa Pesca e Aquicultura, Palmas, TO, Brasil; 6 Embrapa Amazônia Ocidental, Manaus, AM, Brasil

**Keywords:** Management practices, fish parasites, pirarucu, paiche, fish farming, Práticas de manejo, parasitas de peixes, pirarucu, paiche, piscicultura

## Abstract

The objective of this study was to investigate the parasites occurrences and to determine the rates of infestation/parasitic infection in juvenile *Arapaima gigas* from seven fish farms in the state of Amazonas, relating to the characteristics of these fish farms. Of the 70 *A. gigas* evaluated, 43 were infested/infected, with a total of 133 parasites collected. Three fish farms (2, 4, 6) showed the highest levels of prevalence of parasites (100%, 70%, 70%), mean intensity (4.1±2.6, 8.1±9.2, and 2.1±1.3), and mean abundance (4.1±2.6, 5.7±8.1, and 1.5±1.5), respectively. Prevalence ranged from 30% to 100%, mean intensity from 1.0 to 8.1 and mean abundance from 0.3 to 5.7. Monogenea, Digenea, Nematoda (*Hysterothylacium* sp. and *Spirocamallanus* sp.) and Acanthocephala (*Neoechinorhynchus* sp. and *Polyacanthorhynchus* sp.) were identified. The parasites *Hysterothylacium* sp. and *Neoechinorhynchus* sp. were the most prevalent parasites with 31.43% and 15.71%, respectively. The fish presented negative allometry in growth and constant condition factor. Measures to prevent and control the parasitic diseases diagnosed are discussed as well prophylactic practices that contribute to the biosecurity of the farms.

## Resumo

O objetivo deste trabalho foi investigar as ocorrências de parasitos e determinar as taxas de infestação/infecção parasitária em juvenis de *Arapaima gigas* de sete pisciculturas do Estado do Amazonas, relacionando às características dessas pisciculturas. Dos 70 *A. gigas* avaliados, 43 estavam infestados/infectados, com um total de 133 parasitos coletados. As pisciculturas 2, 4 e 6 apresentaram os maiores níveis de prevalência de parasitas (100%, 70%, 70%), intensidade média (4,1±2,6, 8,1±9,2 e 2,1±1,3) e abundância média (4,1±2,6, 5,7±8,1 e 1,5±1,5), respectivamente. A prevalência variou de 30% a 100%, intensidade média de 1,0 a 8,1 e abundância média de 0,3 a 5,7. Monogenea, Digenea, Nematoda (*Hysterothylacium* sp. e *Spirocamallanus* sp.) e Acanthocephala (*Neoechinorhynchus* sp. e *Polyacanthorhynchus* sp.) foram identificados. Os parasitas *Hysterothylacium* sp. e *Neoechinorhynchus* sp. foram os mais prevalentes com 31,43% e 15,71%, respectivamente. Os peixes apresentaram alometria negativa no crescimento e fator de condição constante. Foram discutidas as medidas de prevenção e controle das doenças parasitárias diagnosticadas, assim como práticas profiláticas que contribuam para a biossegurança das fazendas.

## Introduction

Aquaculture in South America plays an important role in food production and, on this continent, and Brazil stands out as the largest producer of freshwater fish. It also has the potential to become one of the largest fish producers in the world (Valladão et al., 2018; Valenti et al., 2021). One species of native fish with great potential for aquaculture is *Arapaima gigas* Schinz, 1822, since it has as remarkable biological aspects such as its rapid growth and air breathing. It also has characteristics that are conducive to breeding such as the possibility of carrying out the feeding transition from live food to commercial feed and the ability to withstand high storage densities in nurseries and during growout phase (Oliveira et al., 2020; Pereira-Filho et al., 2020; Santana et al., 2020; Lima et al., 2021). The state of Amazonas has a number of large-scale fish producing centers. The municipality of Manacapuru is one of these, and it contributes significantly to the production of *A. gigas* (Araújo et al., 2009a; Lima et al., 2015, 2018a; Santana et al., 2017; Valladão et al., 2018; Pereira-Filho et al., 2020).

Despite its potential for rearing, this species faces major problems in its production chain (Lima et al., 2015, 2018a; Valladão et al., 2018; Pereira-Filho et al., 2020). From a sanitary point of view, the main challenge is to reduce the high mortality rate in the fingerling phase, mainly caused by the occurrence of parasitic and bacterial diseases (Morey et al., 2020; Pereira-Filho et al., 2020; Costa et al., 2021; Ferreira et al., 2021; Proietti-Junior et al., 2021). With emphasis on the parasitic diseases, the main groups registered in *A. gigas* are protozoans, myxosporids, monogeneans, digeneans, cestodes, nematodes, acanthocephalans and crustaceans (Morey et al., 2020; Pereira-Filho et al., 2020). Of these, the records of monogeneans, digeneans, nematodes and acanthocephalans in the rearing of *A. gigas* have increased in recent years, and these parasites are pathogenic and some have zoonotic potential (Marinho et al., 2013; Andrade-Porto et al., 2015; Silva et al., 2016; Azevedo et al., 2017; Maciel et al., 2017; Santana et al., 2017; Morey et al., 2019; Ferreira et al., 2021).

In the phylum Platyhelminthes, three classes are found parasitizing *A. gigas*, of which mainly Monogenea and Digenea are highlighted (Maciel et al., 2017; Morey et al., 2019; Serrano-Martínez et al., 2015; Delgado et al., 2007). For Monogeneans, there is the record of three species, *Dawestrema cycloancistrium* Price & Nowlin, 1967, *Dawestrema cycloancistroides* Kritsky et al., 1985 and *Dawestrema punctatum* Kritsky et al., 1985, the first species being the most abundant (Araújo et al., 2009b; Delgado et al., 2013; Marinho et al., 2013; Maciel et al., 2017; Morey et al., 2019). These parasites, found in the skin and gills of fish, have a monoxenic life cycle, with a capacity for high proliferation (Hoai, 2020). High rates of infestation by monogeneans can cause important histopathological changes, such as hyperplasia and fusion of the gill lamellae, which can culminate in fish mortality due to respiratory problems (Tavares-Dias et al., 2021). For digeneans, there is a record of the species *Caballerotrema brasiliense* Thatcher, 1980 and *Caballerotrema arapaimense* Thatcher, 1980 in *A. gigas* (Serrano-Martínez et al., 2015; Delgado et al., 2007). These parasites have a heteroxenic life cycle and, when adults inhabit the intestine, while in the larval phase, metacercariae are considered more aggressive and encyst in the liver, spleen, gills, kidneys, musculature, intestine and eyes (Tavares-Dias et al., 2021).

With regard to nematodes, the species considered to be the most pathogenic in the rearing of *A. gigas* is *Goezia spinulosa* Diesing, 1839 (Santos & Moravec, 2009; Azevedo et al., 2017; Silva et al., 2016, 2017). Other species include *Camallanus tridentatus* Drasche, 1884, *Terranova serrata* Drasche, 1884 (Araújo et al., 2009b), *Nilonema senticosum* Baylis, 1927 (Serrano-Martínez et al., 2015), *Spirocamallanus inopinatus* Travassos, Artigas & Pereira, 1928 (Gaines et al., 2012), *Capillostrongyloides arapaimae* Santos et al., 2008 and larvae of Ascaridoidea gen. sp. (Silva et al., 2016). The larvae of *Hysterothylacium* sp. Ward & Magath, 1917 have zoonotic potential and may cause anisakidosis in humans (Andrade-Porto et al., 2015; Silva et al., 2016; Azevedo et al., 2017). High levels of nematode infections cause severe ulcerative lesions, necrosis in the gastric gland, and inflammatory reactions in the stomach, which can lead to the death of juvenile *A. gigas* (Menezes et al., 2011; Gaines et al., 2012; Azevedo et al., 2017; Miler et al., 2017).

In *A. gigas*, there is a record of two species of acanthocephalans, *Polyacanthorhynchus macrorhynchus* Diesing, 1851 and *Polyacanthorhynchus rhopalorhynchus* Diesing, 1851, the latter occuring in fish of a natural environment (Thatcher, 2006; Santos et al., 2008; Marinho et al., 2013; Santana et al., 2017). However, Silva et al. (2016) reported the occurrence of three genera of the phylum Acanthocephala: *Polyacanthorhynchus* sp., *Neoechinorhynchus* sp. and Acanthocephala gen. sp. in *A. gigas* reared in a semi-intensive system. These parasites have a heteroxenic life cycle and possess a proboscis, which, with its mechanical action, promotes stiffening and thickening of the intestinal wall, thus stimulating an inflammatory process marked by the presence of macrophages, Langerhans cells and lymphocytes, necrosis and tissue degeneration (Jerônimo et al., 2017; Tavares-Dias et al., 2021).

In this context, the growth in the fish farm of *A. gigas* and the intensification of productive systems have contributed to the occurrence and spread of parasitic diseases, especially in the larviculture and the weaning start phase. Therefore, epidemiological studies are of vital importance and must be continuous in order to identify potential risk factors for the occurrence of these diseases. Additionally, the collection of information on infrastructure and management practices adopted in the routine production of young fish must be added to outline practical approaches for the prevention of parasitic diseases in the rearing of *A. gigas*. The objective of this study was to investigate the occurrence of parasites, identify their species and determine the rates of infestation/parasitic infection in juvenile *A. gigas* from seven fish farms in the state of Amazonas, relating to the characteristics of these fish farms, as well as propose prophylactic practices that contribute to the biosecurity of the farms.

## Material and Methods

**Figure 1:**
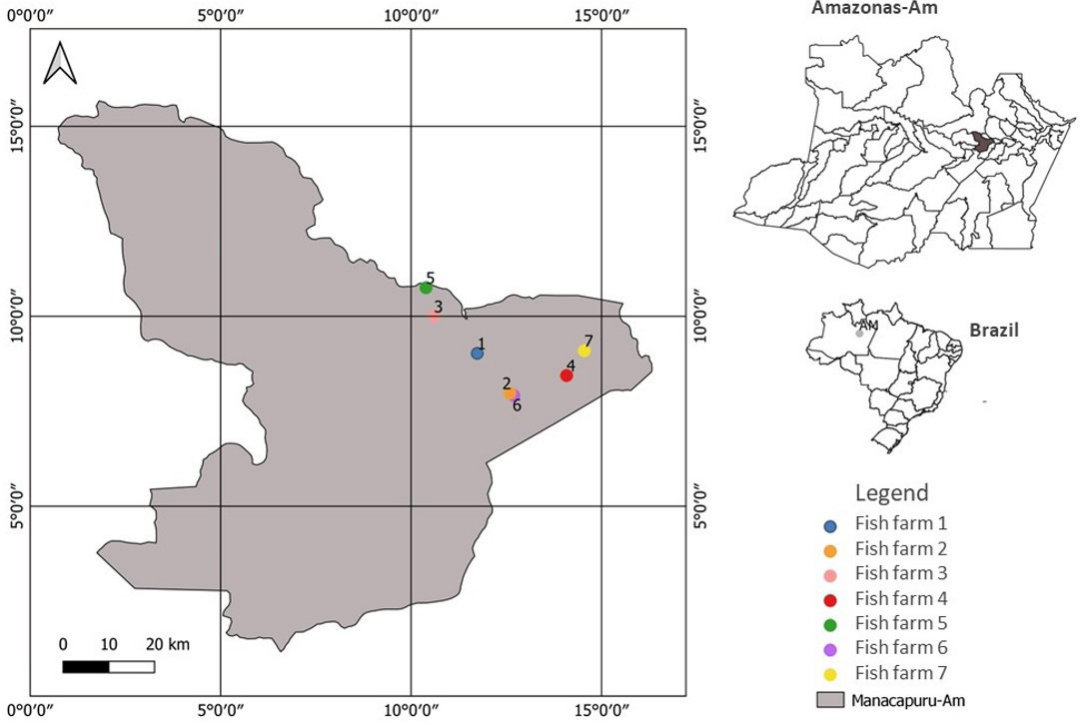
Collection sites of juvenile *Arapaima gigas* in the municipality of Manacapuru, state of Amazonas, Brazil.

A total of 70 juvenile *A. gigas* from 20 to 30 days of age were collected from seven fish farms located in the municipality of Manacapuru, Amazonas state, between March 2020 and March 2021 (Figure 1). These fish farms were characterized as follows: 1) geographical location, 2) breeding structure, 3) flooded area, 4) source of water supply, 5) fish production phase carried out in fish farming, 6) broodstock and 7) fish feeding.

The fish from seven natural spawns that occurred in March, June and July 2020 and January and March 2021 were collected in two points in the farm: 1) directly from the offspring in the ponds where the natural reproductions occurred; 2) from 1,000 L fiberglass tanks were the fish were maintained after the catches of the offspring. After the collections, the fish were transported in a closed system to the Embrapa Amazônia Ocidental laboratory, in Manaus, Amazonas, for parasitological analysis.

During the collection of fish at the seven fish farms, measurements of the water quality, such as pH, temperature and dissolved oxygen were taken with digital devices (pH meter YSI Ambiental 100 and oximeter YSI Pro20, Ohio, USA) and water transparency with a Secchi disc. The alkalinity and hardness of the water were determined by titration with EDTA and methyl orange, respectively, and the total ammonia was measured using the endophenol method (APHA, 1998). The pH was 8.49±1.44, temperature 31.63±0.96 °C, dissolved oxygen 5.40±2.69 mg L^-1^, transparency 56.25±30.09 cm, alkalinity 17.82±10.32 mg L^-1^ CaCO_3_, hardness 16.56±9.62 mg L^-1^ CaCO_3_ and total ammonia 0.44±0.63 mg L^-1^.

In the laboratory, the fish were anesthetized with benzocaine (Honczaryk & Inoue, 2010) in order to record weight (g) and total length (cm), for later calculation of the length-weight ratio and relative condition factor (Kn) (Le Cren, 1951). After biometrics, the fish were euthanized for parasitological evaluation, with analysis of the body, eyes, mouth, opercula, gills and gastrointestinal tract. The collection, fixation, identification and counting of parasites were carried out according to the methodology of Amato et al. (1991), Eiras et al. (2006) and Thatcher (2006). The calculation of parasitic indices of prevalence (P), mean intensity (MI) and mean abundance (MA) was performed according to Bush et al. (1997).

The length-weight relationship was obtained using the equation Wt = *a*. Lt*^b^*, where Wt is the total weight (g), Lt the total length (cm) and *a* and *b* the constants (Le Cren, 1951). The constants were estimated by linear regression of the transformed equation: ln Wt = *ln a* + *b ln* Lt, where *ln* is the natural logarithm. The significance level of *r* was estimated and the value of *b* verified by the *t* test to check that *b* = 0, which is a criterion adopted for classification of the type of growth of the species.

The relative condition factor (Kn) was calculated and then tested with the standard Kn = 1.00, according to Le Cren (1951), using the *t*-test at 5% probability. Analysis of variance was used to compare the differences between weight and total length of fish collected in the fish farms. The Spearman correlation coefficient (*rs*) was used to determine the possible correlation between total weight (g), total length (cm), parasite abundance and relative condition factor. Principal Component Analysis (PCA) was applied to verify a possible relationship between fish farms, biometric data, parasites and relative condition factor (Zar, 1999).

## Results

The production facilities of the seven fish farms selected in this study comprised an area of ponds that ranged from 0.3 to 56.7 hectares (ha), reproductive fishponds from 0.6 to 216 ha and areas with fiberglass tanks of 1,000 L, and 71.4% of the fish farms carried out reproduction, larviculture, the weaning start phase and the first growout phase of *A. gigas* and only one also carried out the second growout phase. In fish farms 1, 2, 5, 6 and 7, reproduction, larviculture, the weaning start phase of *A. gigas* were carried out in ponds and a river dam supplied with water from dammed springs, artesian wells and rivers. Fish farm 4 carried out only the reproduction of *A. gigas* in a pond supplied with water from a dammed spring, while fish farm 3 carried out reproduction, first and second growout phase of *A. gigas* in a pond and river dam supplied with water from a dammed spring (Table 1).

**Table 1:** Geographic coordinates, structure, flooded area, water source, production phase and food offered to breeding and juvenile *Arapaima gigas* fish farms in the municipality of Manacapuru, state of Amazonas.

Fish	Geographical coordinates	Structure	Area (ha)	Water	Phase	Food
1	03º13'54.10”S	Pond and nursery	0.3	Spring and well	Reproduction, larviculture and first growout phase	Breeders: live forage fish; Juveniles: zooplankton, brine shrimp and feed
	60º39'26.62”W		0.6			
2	03°18'37.98”S	Pond	56.7	River	Reproduction, larviculture and first growout phase	Breeders: gutted and frozen forage fish; Juveniles: zooplankton.
	60°35'34.26”W					
3	03º09'31.08”S	Pond and nursery	8	Spring	Reproduction, larviculture, first and second growout phase	Breeders: live forage fish; Juveniles: zooplankton, brine shrimp and feed
	60º44'32.86”W		216.1			
4	03°16'31.90”S	Pond	1.5	Spring	Reproduction and larviculture	Breeders: live forage fish; Juveniles: zooplankton, brine shrimp and feed
	60°28'52.50”W					
5	03°06'07.18”S	Nursery	1.73	Well	Reproduction, larviculture and first growout phase	Breeders: live forage fish; Juveniles: zooplankton, brine shrimp and feed
	60°45'30.10”W					
6	03°18'54.00”S	Pond	56.7	River	Reproduction, larviculture and first growout phase	Breeders: gutted and frozen forage fish; Juveniles: zooplankton
	60°35'07.30”W					
7	03°13'35.40”S	Nursery	2.49	River	Reproduction, larviculture and first growout phase	Breeders: gutted and frozen forage fish and feed; Juveniles: ground fish
	60°26'47.01”W					

In general, the presence of filters or protective screens at the water inlet, continuous monitoring of water quality, control of birds, anti-bird nets and total runoff of water from the ponds were not recorded in the fish farms evaluated, especially those supplied with dammed spring and river water, which consequently does not allow disinfection and fertilization. In fish farm 2, with the retraction of the water levels of the Solimões River, some ponds become empty and others retain water, and some land nurseries have a water supply and drainage system. In fish farm 5, the total emptying of water was recorded, followed by disinfection and fertilization of the land nurseries to stimulate the production of zooplankton that will serve as food for post-larvae and juvenile *A. gigas*.

In the seven fish farms sampled, regarding the breeding stock, it was recorded that *A. gigas* with an average weight of 85 kg were kept isolated and/or maintained with other fish species, such as tambaqui (*Colossoma macropomum*) and matrinxã (*Brycon amazonicus*). The proportion of adult male and female *A. gigas* varied with the type and size of the production facilities and size of the breeders, and were described by fish farmers as having proportions of 1:1 and 3:2, respectively. In the sampled spawns, 2,500 to 3,000 *A. gigas* were caught per spawning, with greater amounts reported in previous offspring.

As for feeding, in fish farms 1, 3, 4, and 5, *A. gigas* breeding stock were fed with live forage fish kept in their reproductive fishponds and river dam. In fish farms 2 and 6, the fish were fed with gutted and frozen forage fish. In fish farm 7, both gutted and frozen forage fish and feed were provided. The feeding of the larvae was initially with the living zooplankton present in the reproductive fishponds. Once caught, fish not sold to fish farmers were transferred to 1,000 L fiberglass tanks used as nurseries. In the nurseries of fish farms 2 and 6, the fish were fed only with zooplankton, while in fish farms 1, 3, 4 and 5, zooplankton and brine shrimp nauplii until the transition to commercial powdered feed containing 45% CP, and in fish farm 7 crushed fish was offered to juveniles *A. gigas* (Table 1).

**Figure 2:**
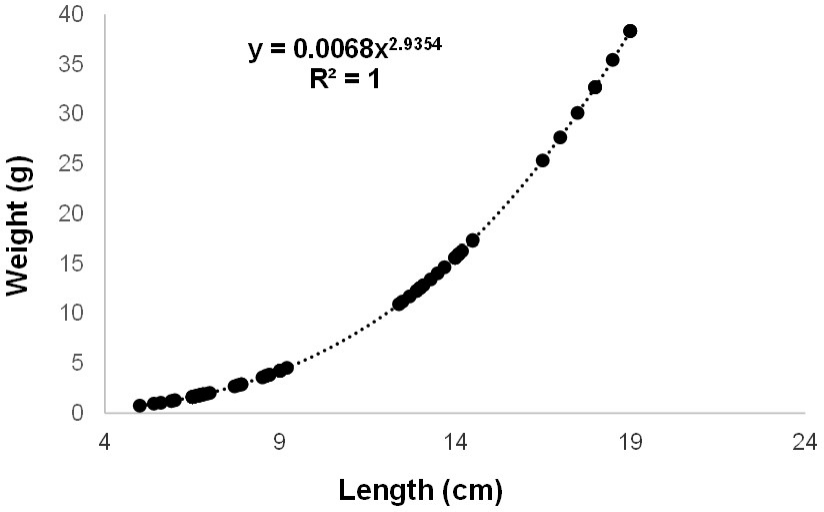
Length-weight ratio of juvenile *Arapaima gigas* collected from fish farms of the municipality of Manacapuru, state of Amazonas.

**Figure 3:**
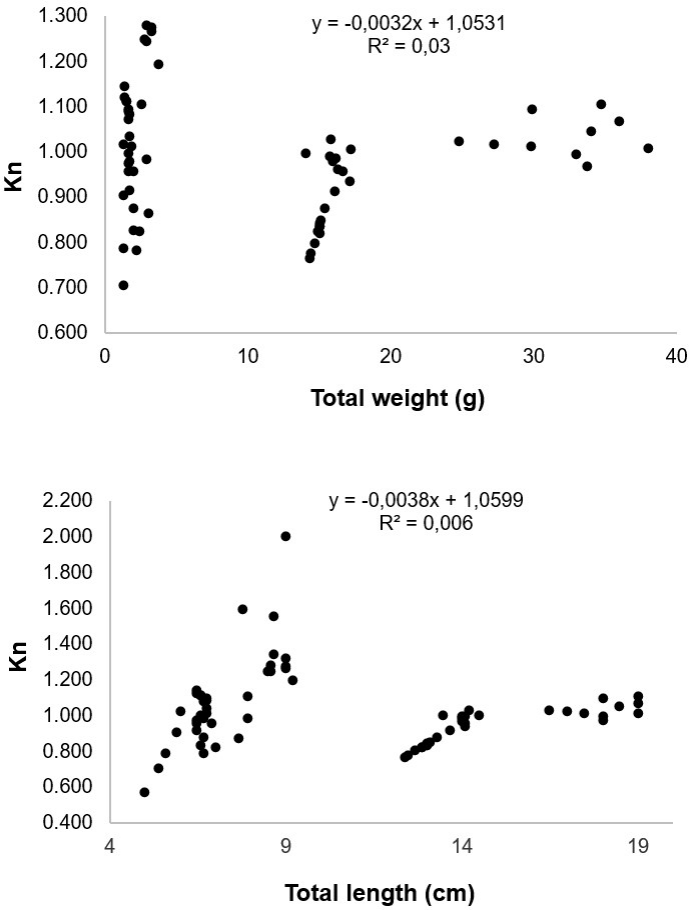
Relationship between the relative condition factor (Kn) and weight (g) and total length (cm) of juvenile *Arapaima gigas* collected from fish farms of the municipality of Manacapuru, state of Amazonas.

Ten specimens of *A. gigas* per brood stock were collected in each fish farm, and the total weight ranged from 0.9 to 38.0 g, the total length ranged from 4.2 to 19.0 cm, and the condition factor ranged from 1.0 to 1.03 (Table 2). The weight and total length of the fish in fish farms 2, 3 and 4 was statistically greater than the other fish farms (p<0.05), with emphasis on fish farm 4 where the fish with larger sizes were collected (Table 2). The fish length-weight ratio equation was Wt = 0.0068*Lt^2.9354^ and r^2^ = 1.00. The value of *b* differed statistically from 3 (p<0.05), indicating that *A. gigas* presents negative allometric growth, and the value of *b* <3 indicates a greater increase in length than in weight (Figure 2). The dispersion diagram of the relation between Kn and weight and total length showed that with growth the condition factor of *A. gigas* juveniles remained constant, indicating good body conditions of the fish collected in the seven fish farms (Figure 3).

**Table 2:** Zootechnical and parasitological indices of juvenile *Arapaima gigas* collected from fish farms in the municipality of Manacapuru, state of Amazonas.

**Fish farm**	**Wt (g) ± SD**	**Lt (cm) ± SD**	**Kn ± SD**	**P (%)**	**MI ± SD**	**MA ± SD**	**N**	**Taxa**
1	1.56 ± 0.14 ^b^	6.54 ± 0.23 ^b^	1.01 ± 0.10	60	1.0 ± 0.0	0.6 ± 0.5	6	Acanthocephala
2	16.15 ± 0.88 ^a^	14.02 ± 0.27 ^a^	1.00 ± 0.01	100	4.1 ± 2.6	4.1 ± 2.6	41	Digenea and Nematoda, Acanthocephala
3	14.93 ± 0.31^a^	12.88 ± 0.27 ^a^	1.00 ± 0.00	40	1.0 ± 0.0	0.5 ± 0.5	4	Nematoda
4	32.13 ± 4.10 ^a^	18.05 ± 0.86 ^a^	1.00 ± 0.01	70	8.1 ± 9.2	5.7 ± 8.1	57	Nematoda
5	3.02 ± 0.47 ^b^	8.83 ± 0.24 ^b^	1.02 ± 0.18	30	1.0 ± 0.0	0.3 ± 0.5	3	Nematoda
6	1.77 ± 0.17 ^b^	6.73 ± 0.47 ^b^	1.03 ± 0.18	70	2.1 ± 1.3	1.5 ± 1.5	15	Monogenea and Nematoda, Acanthocephala
7	2.17 ± 0.66 ^b^	6.65 ± 1.04 ^b^	1.02 ± 0.19	60	1.2 ± 0.4	0.7 ± 0.7	7	Monogenea and Acanthocephala
**Overall mean**	**10.2 ± 10.9**	**10.5 ± 4.2**	**1.02 ± 0.22**	**61.4**	**3.1 ± 4.4**	**1.9 ± 3.8**	**133**	

Wt: total weight; Lt: total length; Kn: relative condition factor; P: prevalence; MI: mean intensity; MA: mean abundance; N: number of parasites collected; SD: standard deviation. a, b: different letters indicate differences between total weight and total length between different fish farms.

The fish were obtained in two spawning periods, five collections from March to July 2020 and two from January to March 2021. Of the 70 fish examined, 43 (61.4%) were infested/infected by 133 parasites. The prevalence ranged from 30 to 100%, a mean intensity of 1.0 to 8.1 and mean abundance of 0.3 to 5.7. The most parasitized fish were found in fish farms 4, 2 and 6, with 57, 41 and 15 parasite specimens, respectively. The least infection occurred in fish farm 5 with three fish infected by one species of parasite (Table 2).

At the class level, trematodes (Digenea) were identified in juvenile *A. gigas*, and at the genus level *Dawestrema* sp. (Monogenea), *Hysterothylacium* sp. and *Spirocamallanus* sp. (Nematoda), *Neoechinorhynchus* sp. and *Polyacanthorhynchus* sp. (Acanthocephala). Monogenea and Digenea had low prevalence (<6%) and only a few specimens were collected (<4%). The nematode *Hysterothylacium* sp. was the most prevalent parasite (31.43%) and was the most collected (58.65%) in this study, which was followed by the acanthocephalans *Neoechinorhynchus* sp., and *Polyacanthorhynchus* sp. and the nematode *Spirocamallanus* sp. (Table 3).

**Table 3:** Prevalence (%), number of parasites collected (N) and frequency of occurrence (F) of parasites in juvenile *Arapaima gigas* collected from fish farms in the city of Manacapuru, state Amazonas.

**Parasite**	**P (%)**	**N**	**F (%)**
*Hysterothylacium* sp. (Nematoda)	31.43	78	58.65
*Neoechinorhynchus* sp. (Acanthocephala)	15.71	39	29.32
*Polyacanthorhynchus* sp. (Acanthocephala)	8.57	6	4.51
Digenea gen. sp.	5.71	5	3.76
*Dawestrema* sp. (Monogenea)	5.71	4	3.01
*Spirocamallanus* sp. (Nematoda)	1.43	1	0.75
**Total**	**68.56**	**133**	**100**

Monogeneans were collected from the gills of the fish from fish farms 6 and 7, and digeneans encysted in the swim bladder of fish, in the form of larval eggs in fish farms 2 and 7, both with low prevalence (Table 4).

**Table 4:** Prevalence (P), mean intensity (MI) and mean abundance (MA) of parasites in juvenile *Arapaima gigas* collected from fish farms in the city of Manacapuru, state of Amazonas. SD: standard deviation.

**Parasite**	**Parasitic indices**	**Fish farm**						
		**1**	**2**	**3**	**4**	**5**	**6**	**7**
*Dawestrema* sp. (Monogenea)	P (%)	**-**	**-**	**-**	**-**	**-**	10.0	30.0
	MI ± SD	**-**	**-**	**-**	**-**	**-**	1.0 ± 0.0	1.0 ± 0.0
	MA ± SD	**-**	**-**	**-**	**-**	**-**	0.10 ± 1.4	0.30 ± 0.7
Digenea gen. sp.	P (%)	**-**	10.0	**-**	**-**	**-**	**-**	**-**
	MI ± SD	**-**	2.0 ± 0.0	**-**	**-**	**-**	**-**	**-**
	MA ± SD	**-**	0.20 ± 2.6	**-**	**-**	**-**	**-**	**-**
*Hysterothylacium* sp. (Nematoda)	P (%)	**-**	10.0	40.0	70.0	30.0	60.0	**-**
	MI ± SD	**-**	1.0 ± 0.0	1.0 ± 0.0	8.1 ± 9.2	1.0 ± 0.0	2.2 ± 1.5	**-**
	MA ± SD	**-**	0.1 ± 2.6	0.4 ± 0.5	5.7 ± 8.2	0.3 ± 0.5	1.3 ± 1.4	**-**
*Spirocamallanus* sp. (Nematoda)	P (%)	**-**	10.0	**-**	**-**	**-**	**-**	**-**
	MI ± SD	**-**	1.0 ± 0.0	**-**	**-**	**-**	**-**	**-**
	MA ± SD	**-**	0.1 ± 2.6	**-**	**-**	**-**	**-**	**-**
*Neoechinorhynchus* sp. (Acanthocephala)	P (%)	**-**	90.0	**-**	**-**	**-**	10.0	10.0
	MI ± SD	**-**	4.1 ± 2.8	**-**	**-**	**-**	1.0 ± 0.0	1.0 ± 0.0
	MA ± SD	**-**	3.7 ± 2.6	**-**	**-**	**-**	0.1 ± 1.4	0.1 ± 0.7
*Polyacanthorhynchus* sp. (Acanthocephala)	P (%)	60.0	**-**	**-**	**-**	**-**	**-**	**-**
	MI ± SD	1.0 ± 0.0	**-**	**-**	**-**	**-**	**-**	**-**
	MA ± SD	0.6 ± 0.5	**-**	**-**	**-**	**-**	**-**	**-**

The nematode parasites of the genus *Hysterothylacium* sp. were collected in *A. gigas* from fish farms 2, 3, 4, 5 and 6, all in the larval stage L_3_, and fish farm 4 had the highest parasitic indices for this genus. A total of 77 larvae were found in the mesentery, one in the stomach and five in the intestine of the fish, thus indicating the mesentery as the main site of infection for these larvae. As for the nematode *Spirocamallanus* sp., the parasitic indices were low, and only one parasite was found in the intestine of a fish (Table 4).

The specimens of *Neoechinorhynchus* sp. were collected in the mesentery and gut of fish from three fish farms, while *Polyacanthorhynchus* sp. was found in the intestines of fish from a single fish farm. In fish farms 2, 6 and 7, the greatest diversity of parasite taxa was observed (Table 2), including the occurrence of the acanthocephalan *Neoechinorhynchus* sp. (Table 4). This finding may be related to a common characteristic among fish farms, which is the water supply of their production facilities that is from river water.

**Figure 4:**
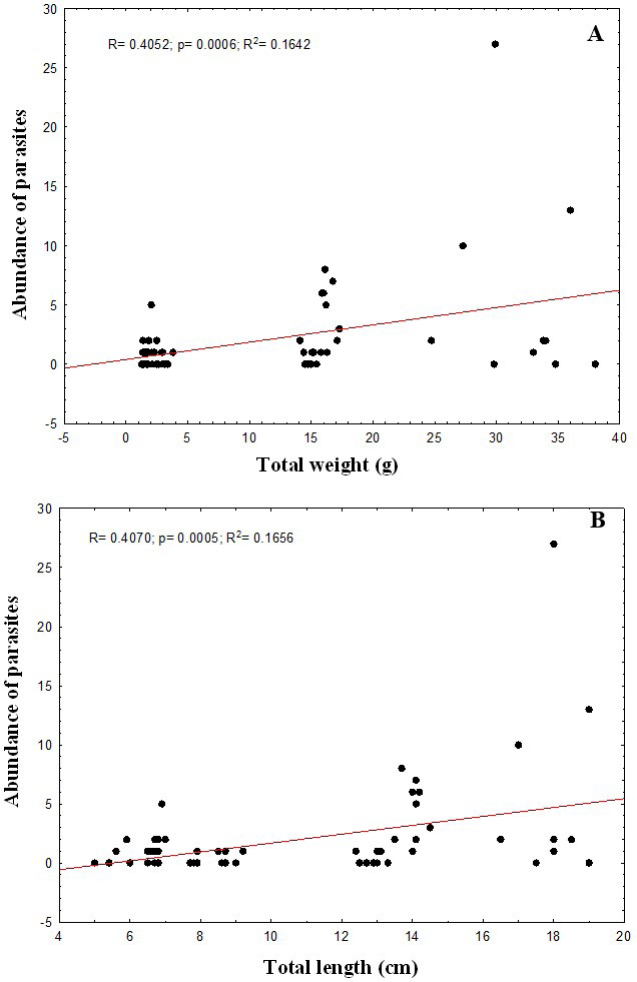
Correlation between abundance of parasites and total weight (g) (A) and total length (cm) (B) of *Arapaima gigas* collected from fish farms in the city of Manacapuru, state of Amazonas.

The correlation between parasite abundance and relative condition factor of fish was not significant (p>0.05). However, the correlations between weight and length and the abundance of parasites were significant (p<0.05). Nonetheless, although positive, the low value of r^2^ explains only 16% of this correlation. The abundance of parasites remained constant and did not influence the growth of *A. gigas* up to the average weight of 10.5±10.9 g and total length of 10.2±4.2 cm (Figure 4).

**Figure 5:**
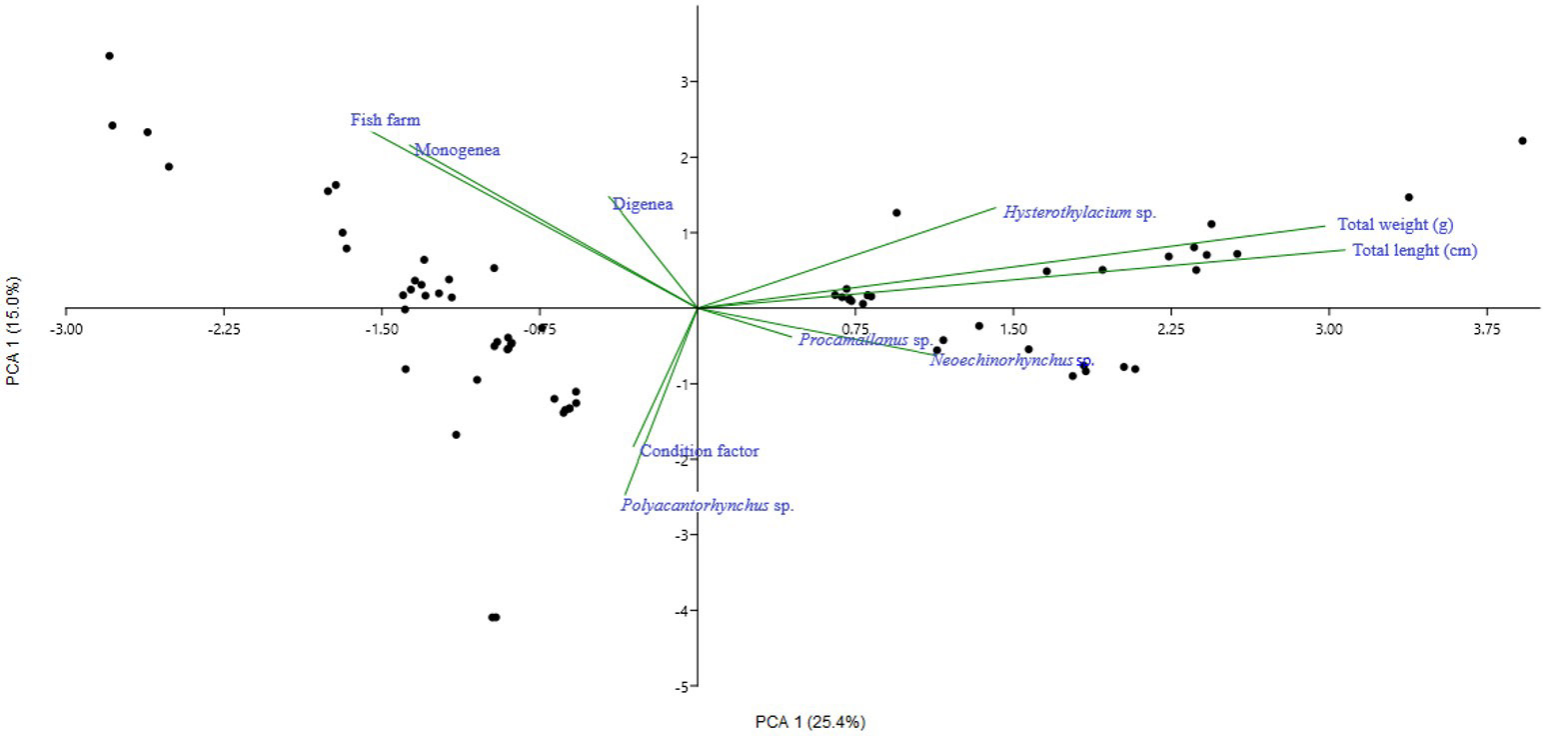
Principal components analysis (PCA) of the data collected from *Arapaima gigas* collected from fish farms in the municipality of Manacapuru, state of Amazonas.

In principal component analysis (PCA) the first two components explain 40.4% of the total variance of the data for *A. gigas*. In axis 1 (PCA 1), a strong and positive relationship was observed between the parasites *Spirocamallanus* sp. and *Neoechinorhynchus* sp. Axis 2 (PCA 2) shows a strong relationship between Monogenea and Digenea parasites and fish farms, but this relationship is negative. For the parasites *Hysterothylacium* sp., *Spirocamallanus* sp. and *Neoechinorhynchus*, in axes 1 and 2, a strong and positive relationship with the weight and length of the fish was also observed. A strong relationship was observed between the condition factor and the acanthocephalan *Polyacanthorhynchus* sp., but this had no additional relationship with any other variable analyzed (Figure 5).

## Discussion

The breeding of *A. gigas* is carried out in four production phases: reproduction larviculture, first and second growout phase. After the reproduction that occurs naturally in the cultivation structures where the breeding stock are kept, juveniles of 2-3 cm are captured and, in the larvicultura and weaning phase, which lasts between 20 to 30 days, they go through the process of food training, which comprises the transition from live food to inert food, until they reach between 10 to 20 cm. In the first growout phase, the juveniles are raised for about 100 days, until they reach a weight between 500 g and 1.0 kg, when they go into the second growout phase. This last phase can last up to a year, when the fish reach about 10 to 12 kg (Lima et al., 2017; Lima et al., 2018b). In this study, five of the fish farms evaluated performed reproduction, larviculture and first growout phase, one performed only reproduction and larviculture, and another also performed the second growout phase of *A. gigas*. As all fish farms carried out larviculture and weaning phase, it is important to highlight the need for a sanitary evaluation of juvenile *A. gigas*, as these can be affected by different pathogens that are capable of compromising the other stages of production.

In this study, the juvenile *A. gigas* were from the natural reproduction of breeding stock raised in ponds and river dam, and were collected directly from the cultivation facilities. These types of breeding structures are used by 93.33% of fish farms in the state of Amazonas and, in the municipality of Manacapuru, only one fish farm has the capacity to produce half a million juvenile *A. gigas* per year, while another can produce juvenile *A. gigas*, *C. macropomum* and *B. amazonicus* up to a quantity of 2 million for the three species (Lima et al., 2019). In the seven fish farms evaluated in this study, 2,500 to 3,000 juvenile *A. gigas* were produced by spawning, with the breeding stock kept isolated and/or maintained with other fish species, at a ratio of males to females of 1:1 and 3:2. Lima (2018) and Rebouças et al. (2014) present the positive results in the reproduction of *A. gigas* in the sex ratio of 1:1. After 20 to 30 days of age, the juvenile *A. gigas* produced in the sampled fish farms are sold throughout the state of Amazonas, mainly in the metropolitan region of Manaus, at the price of R$ 1.00 to R$ 1.50 per centimeter, with their destination being growout phase of the individuals.

The feeding of *A. gigas* breeding stock was done with live forage fish on fish farms 1, 3, 4 and 5, though, in fish farms 2, 6 and 7, it was with gutted and frozen forage fish and, only in fish farm 7, was it also associated with feed for carnivorous fish. The use of forage fish in the diet of breeding stock is due to the absence of commercial feed that meets the nutritional needs of the species and the high cost of feed for carnivorous fish, although the most recommended food for nutrition of these breeders is a mixed diet based on commercial feed (40% CP), with the inclusion of gutted fish and vitamin and mineral supplements (Fonseca & Pereira, 2021).

In relation to juvenile *A. gigas*, fish farms perform the weaning phase mostly with live food, zooplankton and brine shrimp nauplii, with a transition to inert food. The supply of live zooplankton collected in the cultivation facilities for food training can be a source of parasite contamination for juvenile *A. gigas* (Lima et al., 2017; Fonseca & Pereira, 2021). However, the use of previously frozen zooplankton does not impair the growth and survival of juvenile *A. gigas*, and this may be a strategy to minimize the transmission of parasites by oral route in the feeding training phase (Lima et al., 2021). In addition, the inclusion of immunostimulating additives (Aquate Fish ™, 12 g kg^-1^ diet) that promote the improvement in the immunity of juvenile *A. gigas* may be an alternative to increase the defenses of fish against pathogens (Dias et al., 2019). One of the fish farms adopted the use of ground fish to feed the juvenile *A. gigas*; however, Fonseca & Pereira (2021) highlight that this practice is not recommended because it causes poor water quality, the possibility of parasitic problems and inefficiency during training.

As for the source of water supply, some of the fish farms used water from the river that passed near to the fish farms, water from dammed springs or from an artesian well. Water from rivers, as a source of supply for fish farming, can be a source of entry of intermediate hosts in nurseries and ponds, especially if there is no use of filters and screens, which was observed in all the fish farms in this study, thus favoring the development of parasites that have a heteroxenic life cycle. In this study, the fish farms with cultivation facilities that were supplied by river water (2, 6 and 7) presented the highest parasitic indices and the highest parasitic diversity, with occurrence of Monogenea, Digenea, Nematoda and Acanthocephala parasites. Similarly, the fish farms supplied with river water were the only ones that presented the occurrence of the acanthocephalan *Neoechinorhynchus* sp. The physico-chemical parameters of the water of the cultivation facilities, analyzed at the time of collection of the juveniles, were adequate for the rearing of *A. gigas* (Oliveira et al., 2020; Pereira-Filho et al., 2020).

The length-weight relationship is an important tool for estimating the weight of fish when only their length is known, and allows comparisons of fish of the same species from different fish farms; in addition to this information being useful for calculating the condition factor, which allows inferring about the health conditions of fish (Tavares-Dias et al., 2010; Lima et al., 2020). The juveniles sampled in the seven fish farms showed weights ranging from 0.9 to 38.0 g and total length from 4.2 to 19.0 cm, indicating that they were captured from the reproduction structures in different periods after hatching of the eggs, and which explains the statistical difference between weight and length between fish farms. In this study, the growth pattern of juvenile *A. gigas* in fish farms was homogeneous, but the growth presented negative allometry, thus indicating a greater increase in length than in weight. This result corroborates previous studies that explain this same behavior in *A. gigas* in the initial growth phase (Alcântara & Guerra, 1992; Cavero et al., 2003). Conversely, in the second growout phase, *A. gigas* presented isometric growth, as observed in fish of 2.87 kg (Tavares-Dias et al., 2010) and from 23.58 to 52.87 kg (Lima et al., 2020). Isometry in growth is the ideal standard for farmed fish, as it suggests that the increment in weight and length occurs in the same proportion. However, *A. gigas* does not maintain a homogeneous pattern throughout its ontogenetic development, presenting both allometric and isometric growth patterns (Tavares-Dias et al., 2010; Lima et al., 2020).

The growth of fish in terms of length increases with time, but the weight can increase or decrease depending on various factors that can affect the deposition or mobilization of body reserves, and changes the condition factor of the individual and the population (Rêgo et al., 2008). Rebouças et al. (2014) evaluated breeding stock and juvenile *A. gigas* of different sizes in nurseries and did not find differences in the condition factor, which emphasizes the well-being of the fish throughout the breeding period. In this study, the variation in weight and length of *A. gigas* did not affect the condition factor and the Kn value remained constant in the fish belonging to the seven fish farms. The results indicate good health and well-being of juvenile *A. gigas* at this early stage, and suggests that with the low parasitic indices found in this study, until this moment, there was no impairment in fish development. These conditions were also observed in other studies for different fish species such as *C. macropomum* and *B. amazonicus* (Tavares-Dias et al., 2008) and *Rhamdia quelen* (Rossato et al., 2021) in monoculture and Nile tilapia (*Oreochromis niloticus*) in polyculture with shrimp (*Litopenaeus vannamei*) (Brum et al., 2019).

The records of ecto and endoparasites in *A. gigas* farms have increased in recent years, with an emphasis on endoparasites that are pathogenic, including species with zoonotic potential (Andrade-Porto et al., 2015; Silva et al., 2016; Azevedo et al., 2017; Santana et al., 2017; Morey et al., 2020). The diversity of parasites that occur in juvenile *A. gigas* is due to the carnivorous feeding habit of the species, starting with filtration of zooplankton until the predation of molluscs and fish, which increase the chances of infection by endoparasites such as Digenea, Nematoda and Acanthocephala that present a heteroxenic life cycle (Luque & Poulin, 2004; Pereira-Filho et al., 2020; Morey et al., 2020). Another factor is the current condition of natural reproduction of *A. gigas* in which the post-larvae hatch in a shared environment with the parents, where they begin the natural feeding with zooplankton, thus favoring contamination by parasites due to cohabitation and the ingestion of intermediate hosts (Lima et al., 2017).

*Hysterothylacium* sp. (Nematoda: Anisakidae) was the most frequently collected parasite in *A. gigas* (58.65%), all in larval stage 3, and was located in the stomach and intestine, but mainly in the mesentery of fish. Larvae of *Hysterothylacium* sp. can perform migrations between organs inside the host, causing perforations and severe lesions in the stomach and intestine with consequent septicemia, as demonstrated in the fish *Polyodon spathulas* (Miyazaki et al., 1988). Its record in *A. gigas* farms is recent, and was first described by Andrade-Porto et al. (2015), and related to the presence of ascites, lesions and petechiae in the intestinal mucosa of parasitized *A. gigas*. The presence of L_3_ larvae of *Hysterothylacium* sp. in juvenile *A. gigas* indicates that these are intermediate or paratenic hosts of the parasite (Andrade-Porto et al., 2015; Azevedo et al., 2017). The parasitic rates in the five infected fish farms with L_3_ were low, with the highest records in the fish farm 4, where the largest juvenile *A. gigas* (18.05 cm) was collected. Similar indices to the present study were recorded by Andrade-Porto et al. (2015) in *A. gigas* of 14.5±2.1 cm, bred in the municipality of Rio Preto da Eva, Amazonas: P = 98%, MI = 6.02±5.75 and MA = 5.9±5.76. In contrast, Azevedo et al. (2017) found very high rates (P = 98.5%, MI = 75.9±61.6 and MA = 74.8±62) in juveniles of only 5 cm, which caused the mortality of these fish. In the PCA analysis, in the present study, the nematodes *Hysterothylacium* sp. showed a strong and positive relationship with the weight and total length of *A. gigas*, showing that infection occurs in fish of different sizes. The weak positive correlation between *Hysterothylacium* sp. and length of fish found by Azevedo et al. (2017) must have been due to the homogeneous size of the fish. Anisakid nematodes have zoonotic potential and can cause diseases in humans, causing gastrointestinal symptoms and allergic or gastroallergic reactions, and this infection occurs through the consumption of raw or undercooked fish that are parasitized by larvae (L_3_) of nematodes of the family anisakidae (Falla-Zuñiga et al., 2021). Although a member of the same family, records of *Hysterothylacium* sp. infections are rare in humans around the world and are absent in Brazil (Pavanelli et al., 2015).

Specimens of *A. gigas* can be parasitized by different species of nematodes in one or more organs as well as throughout the digestive tract (Santos et al., 2008; Araújo et al., 2009b; Souza & Malta, 2010; Gaines et al., 2012; Serrano-Martínez et al., 2015; Azevedo et al., 2017; Zevallos & Cotrina, 2017; Morey et al., 2020). In this study, the occurrence of *Spirocamallanus* sp. was also recorded in only one fish from one fish farm, with low parasitic indices. This species of parasite has also been reported in approximately 82 species of freshwater fish from Brazil, including *A. gigas*, with low values of mean intensity and abundance, in addition to prevalence ranging from low to moderate, and this low level of parasitism may be, in part, due to its complex heteroxenic life cycle (Neves et al., 2020). Similar to what is observed for *Hysterothylacium* sp., a strong and positive relationship of this parasite with the weight and total length of *A. gigas* was found. However, although the parasitic rates of this study were low, it is important to highlight that high levels of infection by *Spirocamallanus* sp. may cause intestinal obstruction (Rivadeneyra et al., 2020). In addition to histopathological alterations such as areas of focal necrosis, desquamations, inflammatory infiltrate, hemorrhage and cytolysis (Gaines et al., 2012), which reinforces the need for constant monitoring of parasitic fauna in fish.

*Neoechinorhynchus* sp. was the second most collected parasite, and the common characteristic among the three fish farms that recorded its occurrence was the supply of the cultivation facilities with river water, which is a condition that may have contributed to the entry of intermediate hosts contaminated with this acanthocephalan. The ostracod *Cypridopsis vidua* has been identified as an intermediate host of *N. buttnerae*, which is a parasite of *C. macropomum* (Lourenço et al., 2017), and was recorded in fish farms contaminated by this parasite (Chagas et al., 2019). *Neoechinorhynchus buttnerae* currently represents a serious health problem in the rearing of *C. macropomum*, mainly in the northern region of Brazil, with significant economic losses in fish production (Silva-Gomes et al., 2017; Chagas et al., 2019; Valladão et al., 2020). However, little information exists for acanthocephalans in *A. gigas*. This is the second report of acanthocephalans of the genus *Neoechinorhynchus* in *A. gigas* farms. In the first case, reported by Silva et al. (2016), only one specimen was collected in the intestine and, in this study, *Neoechinorhynchus* sp. was found in the intestine and mesentery. A strong and positive relationship between these parasites and the weight and total length of *A. gigas* was observed. In addition to a strong relationship between the acanthocephalan *Neoechinorhynchus* sp. and the nematode *Spirocamallanus* sp., which suggests that they have intermediate hosts in common, both being present in the supply water and in the live feeding route of *A. gigas* fry.

It is noteworthy that until recently the only acanthocephalan of *A. gigas* in captivity in Brazil was *P. macrorhynchus* (Marinho et al., 2013). However, Silva et al. (2016) identified, in addition to the genus *Polyacanthorhynchus* sp., the genera *Neoechinorhynchus* sp. and Acanthocephala gen. sp., emphasizing that *A. gigas* bred in semi-intensive systems can be parasitized by a diversity of acanthocephalans. In this study, the Acanthocephala *Polyacanthorhynchus* sp. was recorded in only one fish farm in the city of Manacapuru, and was the third most frequent parasite, but with low intensity and medium abundance. Similarly, *P. macrorhynchus* was recorded by Santana et al. (2017) with low parasitic indices, P = 20%, MI = 1.25 and MA = 0.25, and was attributed by the authors to transmission due to the feeding of *A. gigas* with fish-based feeds. Considering the relevance of infection by acanthocephalans in fish farming of native fish, it is urgent and necessary to identify and describe the acanthocephalan species that affect farmed *A. gigas*, in order to contribute to the development of strategies for the prevention and control of this parasitosis.

Digenea and *Dawestrema* sp. (Monogenea) presented the fourth and fifth frequency of occurrence, respectively, among the diversity of groups of parasites identified and, according to the PCA analysis, a positive but weak relationship was observed between fish farms and these parasites. Digeneans were found encysted in the swim bladder of juvenile *A. gigas* in only two fish farms, with low parasitic rates. There is a report of the occurrence of *C. brasiliense* in the intestine and stomach wall of adult *A. gigas*. On the one hand, Serrano-Martínez et al. (2015) found low parasitic indices (P = 6.7%, MI = 2.0 and MA = 0.1) in *A. gigas* over one year of age and 2.0 kg; however, on the other hand, Delgado et al. (2007) found high parasitic indices (P = 75%, MI = 28.3 and MA = 21.5) in *A. gigas* weighing 64.9 kg. These flatworms have two suction cup-like attachment organs that cause significant damage when they are encysted in the various organs of the fish, with the exception of the intestine where the damage is localized in the intestinal lumen and results from the attachment and feeding of the parasite (Dezfuli et al., 2016).

Monogeneans are parasites that proliferate rapidly due to their monoxenic life cycle, and can occur mainly on the body surface, gills and nostrils of fish. These attach themselves through the haptor located in the posterior region of the body (Hoai, 2020). High infection rates can cause important blood and histopathological alterations, and culminate in fish mortality due to respiratory problems (Tavares-Dias et al., 2021). In farmed *A. gigas,* two species are reported, *D. cycloancistrium* and *D. cycloancistroides*, the first species being more abundant (Delgado et al., 2013; Marinho et al., 2013; Maciel et al., 2017; Morey et al., 2019). In this study, monogeneans had a low frequency of occurrence and parasitic indices in juvenile *A. gigas* of 3.01%, when compared to the values recorded by Morey et al. (2019) and Maciel et al. (2017). Marinho et al. (2013) reported a prevalence of 70 and 100%, mean intensity of 57.3 and 214.7 and mean abundance of 40.1 and 214.7 for the species *D. cycloancistrium* and *D. cycloancistroides* in juvenile *A. gigas* of 15 g from two fish farms in the state of Amapá. These authors reported a negative correlation between the condition factor and the number of monogeneans, indicating the pathogenicity of these parasites when high parasitic indices are encountered, though this was not observed in this study due to the low levels of infestation.

The juvenile *A. gigas* raised in seven fish farms in the municipality of Manacapuru, state of Amazonas, were parasitized by at least one species of parasite, but the abundance of these organisms was considered low, which reflected in the maintenance of the condition factor of the animals. Unlike other studies, the specimens of *A. gigas* were mainly affected by endohelminths and did not present symptoms. The pathogenesis of these parasites is dependent on the species, intensity of infection, affected organ or tissue and intensity of histopathological damage, among other factors (Dezfuli et al., 2016). In this sense, attention is required regarding the water supply on the property, the feeding of the breeding stock and general sanitary management of juvenile *A. gigas* after capture in the reproduction facilities. Finally, the results obtained through this research reinforce the need for constant monitoring of fish in breeding environments, and the appropriate diagnosis for the establishment of measures to prevent and control parasitic diseases and, thus, avoid high mortality rates of *A. gigas* in the fry phase, one of the major obstacles in the rearing of this species.
